# Review of recent advances in frequency-domain near-infrared spectroscopy technologies [Invited]

**DOI:** 10.1364/BOE.484044

**Published:** 2023-06-12

**Authors:** Xinkai Zhou, Yunjia Xia, Julie Uchitel, Liam Collins-Jones, Shufan Yang, Rui Loureiro, Robert J. Cooper, Hubin Zhao

**Affiliations:** 1 HUB of Intelligent Neuro-engineering (HUBIN), Aspire CREATe, IOMS, Division of Surgery and Interventional Science, University College London (UCL), London, HA7 4LP, UK; 2 DOT-HUB, Department of Medical Physics & Biomedical Engineering, UCL, London, WC1E 6BT, UK; 3 Department of Paediatrics, University of Cambridge, Cambridge, CB2 0QQ, UK; 4 School of Computing, Engineering & Build Environment, Edinburgh Napier University, Edinburgh, UK; 5 Aspire CREATe, Department of Orthopaedics & Musculoskeletal Science, UCL, London, HA7 4LP, UK

## Abstract

Over the past several decades, near-infrared spectroscopy (NIRS) has become a popular research and clinical tool for non-invasively measuring the oxygenation of biological tissues, with particular emphasis on applications to the human brain. In most cases, NIRS studies are performed using continuous-wave NIRS (CW-NIRS), which can only provide information on relative changes in chromophore concentrations, such as oxygenated and deoxygenated hemoglobin, as well as estimates of tissue oxygen saturation. Another type of NIRS known as frequency-domain NIRS (FD-NIRS) has significant advantages: it can directly measure optical pathlength and thus quantify the scattering and absorption coefficients of sampled tissues and provide direct measurements of absolute chromophore concentrations. This review describes the current status of FD-NIRS technologies, their performance, their advantages, and their limitations as compared to other NIRS methods. Significant landmarks of technological progress include the development of both benchtop and portable/wearable FD-NIRS technologies, sensitive front-end photonic components, and high-frequency phase measurements. Clinical applications of FD-NIRS technologies are discussed to provide context on current applications and needed areas of improvement. The review concludes by providing a roadmap toward the next generation of fully wearable, low-cost FD-NIRS systems.

## 1. Introduction

Near-infrared spectroscopy (NIRS) is a non-invasive optical sensing technique that uses light within the near infrared (NIR) range (650 to ∼1000 nm) to measure changes in the optical properties of an object [1]. NIRS can be applied to study biological tissue, which exhibits relatively low absorption within the NIR range. By measuring the change in light intensity, change in phase, or the time it takes photons to travel between a NIRS source and a detector, the optical properties of a tissue can be investigated [2–4]. NIRS has been applied in a variety of research and clinical care settings, including neonatal care [5,6], breast cancer imaging [7], muscle training [8], and neurocritical care, among others [9,10].

To date, the few clinically established NIRS methods are based on continuous-wave (CW) devices (i.e., those which provide continuous optical illumination) with a configuration that permits spatially-resolved spectroscopy. Spatially resolved spectroscopy (SRS) can be used to estimate absolute tissue oxygen saturation, which is a highly valuable clinical parameter [11] However, SRS is based on a number of assumptions (including assumptions pertaining to consistent skin coupling and to the relationship between scattering coefficient and wavelength) that can affect its reliability. All CW-NIRS only assesses changes in light intensity and therefore require assumptions to be made about optical scattering. This is one reason why CW-NIRS approaches cannot provide absolute measures of the concentration of absorbing molecules such as oxyhaemoglobin and deoxyhaemoglobin. Instead, by assuming scattering to be constant over the course of an experiment, CW-NIRS measures the *changes* in hemoglobin concentrations in the brain over time, or between periods or control and experimental stimuli. CW-NIRS has therefore been widely applied in studies of functional brain activation in a technique known as functional NIRS (fNIRS) [3]. However, even measures of changes in chromophore concentration over time using CW-NIRS require an estimate of optical pathlength, which introduces another significant source of error [12].

Time-resolved NIRS in both the frequency-domain (FD-NIRS) and time-domain (TD-NIRS) [13] can provide advantages over traditional CW-NIRS. These methods use time-dependent source illumination schemes that can separate the effects of optical absorption from those of optical scattering, and can provide absolute measurements of the absorption and scattering coefficients of the interrogated tissues. Unlike CW-NIRS, FD-NIRS uses a modulated NIR light source and then measures both the detected light intensity attenuation and the phase shift of that modulation. An estimation of the scattering parameters can be experimentally determined from the phase shift, providing a more accurate estimation of the scattering properties of tissue for a given subject [14,15]. The use of a phase shift measurement in FD-NIRS allows for quantification of chromophore concentrations, as well as comparisons of subjects cross-sectionally and longitudinally [16]. FD-NIRS could also potentially provide improved imaging quality and spatial resolution relative to CW-NIRS [15].

The development of FD-NIRS approaches has significant implications for clinical applications of NIRS methods. FD-NIRS has the potential to provide precise measurements f absorption and scattering coefficients with superior depth specificity, which will translate to more accurate and reliable measures of cerebral tissue oxygenation. However, the development of clinically compatible FD-NIRS instrumentation has been impeded by its poor scalability as well as its relatively complex and slow operation [15]. Significant improvements are needed in current FD-NIRS technologies before it can replace or rival CW-NIRS in clinical contexts.

In this article, we present an in-depth review of the current status and future directions of FD-NIRS technologies. While a previous review on this topic has primarily focused on the theory of FD-NIRS and its general applications [2]; here, we aim to provide an analysis of FD-NIRS hardware, limitations and areas where improvement is needed, and current clinical applications. The remainder of this article is organized as follows: Section 2 reviews the key developments of FD-NIRS technologies to date and describes the principal characteristics of current systems. Section 3 explores the current status of the key components of these technologies, including light sources, detectors, and phase measurement units. Section 4 summarizes the current clinical applications using FD-NIRS. Finally, Section 5 summarizes the key points of the article with a discussion of future advancements.

## 2. State-of-the-art, FD-NIRS technologies

### 2.1 Search strategy for FD-NIRS technology publications

Our search strategy for articles to be included in this review was as follows: the Google Scholar and Web of Science search engines were used for keyword searches [(frequency-domain OR FD) AND (near-infrared spectroscopy (NIRS) OR fNIRS OR diffuse optical spectroscopy OR DOS]. Results were then manually screened to determine whether the work described technology development of FD-NIRS systems in the past 20 years. This search strategy resulted in 53 key publications, the details of which are summarized in Table 1. These papers include 24 groups of publications that describe different developmental stages of the same technology. In these cases, the quoted characteristics in Table 1 refer to the most recent available information.

**Table 1.** Characteristics of Key FD-fNIRS Technologies.

Author, Year	Type	No. of Sources/Detector locations	No. of channels	Wavelength (nm)	Source Power (mW)	Source-Detector Separation (mm)	Dynamic range (dB)	Modulation frequency (MHz)	Phase resolution (degree)	Noise Equivalent Power
ISS Imagent [17]	Benchtop	128/32	512	690, 830	10	-	-	110-400	-	-
Campbell et al., 2020 [21]	Benchtop	1/1	1	631, 660, 689, 782, 828, 849	-	14 - 30	-	20 - 460	-	-
Choe et al., 2009 [23]	Benchtop	9/4	-	690, 750, 786, 830	-	-	-	70	-	-
Zhang et al., 2005 [24,25]	Benchtop	9/1	9 × 40	685, 830	-	-	60	70	0.25	1 pW /√Hz
El-Ghussein et al., 2013 [27]	Benchtop	16/16	256	660, 735, 785, 808, 826, 849	-	-	100	100	0.4 - 0.5	-
Carp et al., 2017 [29]	Benchtop	4/1	4	670, 690, 700, 730, 760, 780, 810, 830	2-5	15 - 30	-	110	-	-
Mackey et al., 2020 [30]	Benchtop	4/12	48	685, 830	10-14	20 - 35	-	100	-	-
Lee et al., 2022 [31]	Benchtop	-	-	785, 824	-	-	-	20, 30, 40	-	-
Huang et al., 2017 [32]	Benchtop	1/1	1	700, 750, 800, 1030	-	-	-	54.77 -985.86	-	-
Zimmermann et al., 2016 [33]	Benchtop	20/1	20 × 24	685, 830	50	-	>115	67.5, 75	0.172	<1.4 pW /√Hz
Thompson et al., 2021 [34]	Benchtop	12/6	72	692, 850	12.5	-	-	-	-	-
Wathen et al., 2021 [36]	Benchtop	32/32	1024	690, 852	12.5	13 - 40	∼60	211	-	20.5 fW / √Hz
Chen et al., 2004 [40]	Portable	1/1	1	660, 780, 830	-	-	85	140	-	-
O’Sullivan et al., 2017 [42]	Portable	1/1	1	680, 795, 850	11.6-17.8	20	-	50 - 1000	-	-
Torjesen et al., 2017 [46]	Benchtop	1/1	1	658, 690, 785, 808, 830, 850	-	30, 10	-	50 - 400	0.38 (50 MHz), 1.7(Avg)	-
Istfan et al., 2019 [50]	Wearable	1/1	1	660, 680, 775, 795	-	13	54.4	50 - 500	-	-
Applegate et al., 2021 [51]	Portable	1/1	1	690, 730, 785, 808, 830, 850	1.2-7.84	20	-	50 - 500	-	-
Stillwell et al., 2021 [58]	Portable	1/1	1	690, 785, 808, 850, 940 980	-	15 - 30	70	1 - 400	0.227 - 0.557	-
Miao et al., 2018 [63]	Wearable	1/1	1	685, 785, 830	35-100 (Factory)	20 - 25	33	80	0.2	3.2 pW / √Hz
Kılıç et al., 2022 [64]	Wearable	1/4	4	690, 830	2.5	20	60	80	0.35	-
Yazdi et al., 2017 [65]	Portable	1/1	1	690, 785, 830, 980	-	15, 20, 25	33	50-1000	0.1 (100 MHz), 2(400 MHz)	-
Chen et al., 2022 [66]	Wearable	1/1	1	685	35-50, (Factory)	-	60	10	0.006	-
Koh et al., 2022 [67]	Wearable	1/1	1	-	-	-	-	<150	<1.4	-
Scammon, 2022 [69]	Portable	1/1	1	785	25 (Factory)	10	-	103.55-327.93	-	-

While the technologies described in these works vary significantly, we have attempted to classify these systems into two broad categories on the basis of their form factors. The technologies presented in Refs. [14,17–39] were developed as benchtop devices using somewhat bulky control electronics and fiber bundles. We categorized these technologies as “benchtop technologies”. In contrast, miniaturized electronics and/or integrated optical components were implemented in many other publications [40–69] in an effort to achieve a fibreless and/or lightweight device with a small footprint. These technologies are referred to as “portable or wearable technologies”.

### 2.2 Benchtop technologies

A key milestone in the development of FD-NIRS devices was the production of the first commercial multi-channel FD-NIRS device, the Imagent system (ISS, US) [17]. The Imagent System was first demonstrated in [70,71], but continues to be employed by numerous labs today. The Imagent system consists of up to 64 laser diode-based light sources (690 and 830 nm) that are typically modulated at a frequency of 110 MHz. A driver circuit controlled by a computer is utilized to control the output optical power of the light sources. The driver circuit enables the system to switch the activated light sources every 20 ms. Optical detection is provided by up to 32 PMTs. The system weighs ∼20 kg with a size of 46 × 43 × 23 cm^3^. The Imagent system therefore has the capacity for relatively high channel count acquisition, and has the potential for real-time monitoring of tissue optical properties. However, the limited portability of the system does limit its utility. Despite this, the Imagent FD-NIRS system has been applied in a wide range of clinical and neuroscientific applications over many years [72–74].

Another notable early development was described in 2000, when Bevilacqua et al. [18] combined the principles of CW-NIRS and frequency domain photon migration (FDPM) to construct a FD-NIRS system. The system applied a commercial network analyzer (Hewlett-Packard 8753C, US) to obtain a frequency-domain measurement. Seven amplitude-modulated laser diodes (672, 800, 806, 852, 896, 913, and 978 nm) were implemented as light sources. Light was detected by an avalanche photodiode (APD) unit (C556P-56045-03, Hamamatsu, Japan). The network analyzer was used to modulate the light at 251 different frequencies ranging from 100 to 700 MHz, as well as measure the phase and amplitudes of the output signal from the APD. Of note, although the work [18] published in 2000 is not in the scope of “in past 20 years”, due to its clear relevance to the work [19] published in 2017, we decided to still include the work [18] into discussions as a “special case”.

**Fig. 1.**
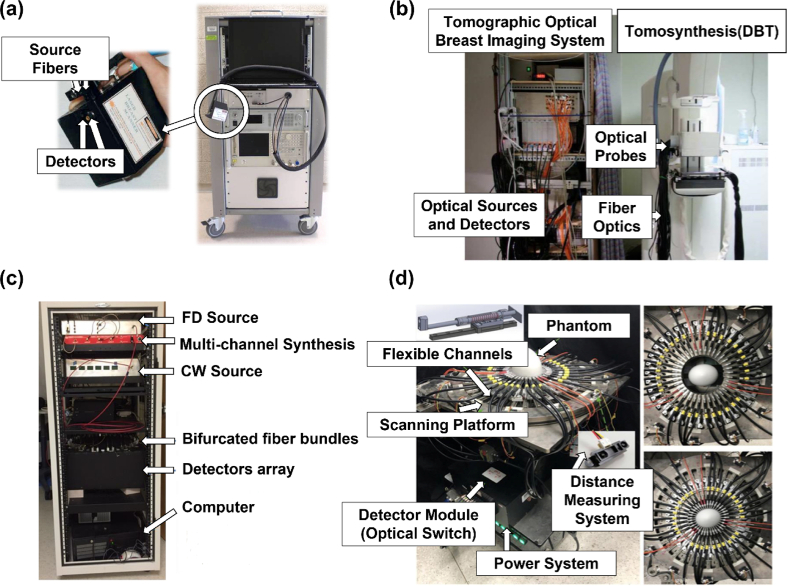
Examples of some benchtop-based FD-NIRS systems. (a) The NIRS device described by O’Sullivan et al. The subfigure on the left demonstrates the handpiece placed in contact with the tissue. This figure is taken with permission from [14,19]. (b) The combined Tomographic Optical Breast Imaging (TOBI)/Tomosynthesis (DBT) system described by Fang et al. The TOBI system includes both RF and CW source/detector modules and the fiber optics interface attached to the tomosynthesis system on the right. This figure is taken with permission from [25]. (c) The FD + CW NIRS system for breast cancer diagnosis described by Zhao et al. This figure is taken with permission from [28]. (d) The NIRS system with flexible optical channels for breast tumor detection described by Lee et al. The subfigure on the right demonstrates the maximal (170 mm) and minimal (60 plus 20 mm flexible range) measuring dimensions. This figure is taken with permission from [31].

A similar approach was pursued much later in 2012, when O’Sullivan et al. [14] implemented a new commercial radiofrequency (RF) network analyzer (Agilent 8753E, Santa Clara, US). A handpiece (Fig. 1(a)) that contacted the tissue was also utilized to separate the source fiber ends away from an integrated high-speed APD (C5658 with S-6045-03 APD, Hamamatsu, US) to a set distance (adjustable from 22 to 34 mm).

In 2017, Leproux et al. [19] demonstrated the system performance in an application to breast imaging. Six laser diodes at the wavelengths of 660, 680, 785, 810, 830, and 850 nm, modulated at 601 modulation frequencies from 50 to 600 MHz, were used as the light source, while the rest of the system was the same as the prior version developed in [14]. In the same year, Peterson et al. [20] applied the system for the measurement of bone sarcoma. The light source wavelengths were then modified (631, 660, 689, 782, 828, and 849 nm) by Campbell and O’Sullivan [21]. The system [21] was also modified such that the source-detector separation (SDS) could also be adjusted via a translating stage (Thorlabs LTS300, Newton, US).

These systems in [14,18–21] were able to quantify the broadband optical absorption of the tissue. Source fibers and detectors located on the same handpiece provided a single measurement channel (i.e. source-detector pair) at each wavelength with an adjustable SDS [19,21]. The system, however, also had some limitations. The signal-to-noise ratio (SNR) of some data collected at high modulation frequencies and long SDS (>28 mm) was relatively low (only ∼6 dB at a wavelength of 659 nm), affecting the measurement accuracy of optical properties and chromophore concentrations [20]. Moreover, although the system was fitted with wheels to provide some portability, it was still somewhat bulky, thus limiting the range of environments in which it could be applied.

In 2003, Culver et al. [22] developed a benchtop, parallel-plate hybrid FD/CW Diffuse Optical Tomography (DOT) system for breast imaging. DOT is an extension of NIRS that utilized high numbers of NIR light sources and detectors to provide overlapping spatial sampling of the target object, which can yield 3D images [75]. In this system, four laser diodes (690, 750, 786, and 830 nm) modulated at a frequency of 70 MHz were applied as light sources. The source output was then optically switched to one of 45 source fibers. Optical detection was provided by nine APDs (C5331-04, Hamamatsu, Japan) for FD measurements. An in-phase and quadrature-phase (I/Q) demodulator (ZFMIQ-70D, 70 MHz, Mini-Circuits, US) and a low pass filter were then applied to extract the phase and amplitude of the output current from APDs. In 2009, Choe et al. [23] in the same group added two CW lasers at 650 and 905 nm to improve the separation of chromophore contributions, while the FD component of the system remained unchanged.

The works in [22,23] successfully combined the FD and CW components in a DOT system. For the FD measurement, the system achieved a noise equivalent power (NEP) of $3pW/\sqrt{Hz}$ , a linearity in the amplitude of 1% and phase drift of ${\mathrm{0.25}}^{\circ }$ over 80 dB [22]. In addition, more light source positions could be applied to improve the sampling density of the tissue. However, the number of effective FD-measurement channels remained unclear.

In 2005, Zhang et al. [24] proposed an imaging system that combined DOT with digital X-ray tomosynthesis for breast tissue imaging. Two lasers (785 and 830 nm) modulated at a frequency of 70 MHz were utilized as optical sources. The light was selected by an optical multiplexer and then switched to 40 different positions. The light emitted from each position could be detected by 8 detectors, thus up to 320 optical channels at each wavelength could be generated. Emerging light was first detected by APDs (C5331-04, Hamamatsu, Japan), and then converted and demodulated with I/Q demodulation methods. Two 16-bit analogue-to-digital converter (ADC) boards were employed to sample the demodulated voltages in which the amplitudes and phases were calculated, respectively. In 2009, Fang et al. [25], from the same research group, modified the above-mentioned FD-NIRS system [24] with a new 685 nm RF laser (replacing the 785 nm laser) to optimize the RF laser wavelength spacing, while the overall architecture of the RF imaging system remained the same (see Fig. 1(b)). This system achieved a dynamic range of 60 dB, a NEP of $1pW/\sqrt{Hz}$ , and a 12 Hz single-channel sampling rate.

Several research groups have explored the use of photo-multiplier tubes (PMTs) to enhance the performance of FD-NIRS systems. In 2001, McBride et al. [26] presented a FD-NIRS system for breast imaging based on PMT detectors. For the light sources, an RF signal was utilized to drive five intensity-modulated laser diodes (660, 761, 785, 808 and 826 nm) at 100 MHz. The light was then directed to an optical switch for multiplexing of the position of sources and detectors. Sixteen PMT (H9305-3, Hamamatsu, Japan) were employed to detect the light. This provided a total of 256 channels (16 sources × 16 detectors) at each wavelength. The output was further down-converted and collected by the Data Acquisition (DAQ) board. The measurements of different wavelengths were conducted in sequence, resulting in a measurement time of up to 20 minutes. In 2013, El Ghussein et al. [27] added an amplifier after each PMT and a programmable gain amplifier to the DAQ board to improve the SNR by a factor of 1000. In 2016, Zhao et al. [28] improved the system (Fig. 1(c)) measuring speed by utilizing a three-wavelength FD source module which consisted of three laser diodes (661, 785 and 826 nm) with signal generated from a multi-channel RF synthesizer (HS2004, Holzworth Instruments, US), the system achieved simultaneous wavelength acquisition. These works [26–28] successfully utilized PMTs as detectors for FD measurements, achieving a dynamic range of 100 dB. However, the high bias voltage of PMT (∼ 1000 V) would be a concern for safety and power consumption. In addition, the sensitivity of the PMT decreased rapidly at wavelengths over 850 nm, limiting the implementation of sources at longer wavelengths.

In 2017, Carp et al. [29] described a commercially available instrument named the “MetaOx” which also implemented PMTs as detectors. The system integrated FD-NIRS and diffuse correlation spectroscopy (DCS) measurements. For the FD-NIRS component, the instrument provided one multi-wavelength source and four detectors. Eight individual laser diodes (670, 690, 705, 730, 760, 780, 810, 830 nm) were used as sources and modulated at 110 MHz (adjustable) via a direct digital synthesis system (DSS), with an average power of 2-5 mW. The modulated light was coupled into an 8-into-1 fiber bundle. Four PMTs (R9880U-01, Hamamatsu, Japan) were used as detectors, providing 4 optical channels with an SDS of 15-30 mm at each wavelength. The output of the PMTs was digitized via a 16-bit DAQ card (Daq3000USE, IOTech, UK) at a sampling rate of 1 MHz. Channel-by-channel calculations were conducted to determine the AC and DC amplitudes of the signal as well as its phase change values.

In 2020, Mackey et al. [30] designed a FD-fNIRS system which also utilized PMTs as detectors. An electronic control and a data acquisition system were developed to control the modulation signal and receive signals from the PMTs. Two lasers were coupled into polarization-maintaining optical fibers to modulate the light at frequencies from 1 Hz to 120 MHz via a commercial function generator. The modulated light was then combined and switched to 12 source-fibers. Detection was provided by four high-speed PMTs, yielding a total of 48 optical channels with an SDS of 20–35 mm at each wavelength. The signal from the PMTs was down-converted from 100 MHz to 500 Hz. These signals were then digitized and delivered to a Raspberry Pi to control data storage. In this work, the implementation of the electronic control and data acquisition system greatly reduced the processing time and cost of the system. However, the portability of the system was relatively limited.

In 2022, Lee et al. [31] demonstrated a FD-NIRS system with two measurement schemes for breast tumor detection. One scheme implemented combined source modulation (combining 20, 30 and 40 MHz source power) and an individual frequency shift Δf to modulate the laser diode. The light was detected by a PMT, then amplified and mixed with the combined sinusoids to obtain low-frequency signals. The amplitude and phase of the mixed signals were then singled out from a varied Δf , achieving the simultaneous measurement of light at three different wavelengths. The other scheme used flexible channel positioning as shown in Fig. 1(d). The light sources were arranged in an oval shape and the position of each light source could be adjusted within a fixed distance by means of a designed mechanical structure.

This work [31] successfully employed two measurement schemes to improve the reconstructed image quality. The measurement time was shortened using simultaneous multiple sinusoids driving light sources. This also reduced the cross talk between absorption and scattering coefficients. The flexible-channel scheme minimized the potential geometrical mismatch of the measurement when measuring different shapes of the breast. Some limitations, however, persist with this system.

In addition to the new detectors, investigations have also been carried out on new types of light sources. In 2017, Huang et al. [32] proposed a wavelength-tunable, ultra-broadband light source for FD-NIRS system. A compact Yb-YAG laser (Mikan, Amplitude System, France) was implemented as the light source, with a 1.03 μm central wavelength, a 250-fs pulse width and a 54.77 MHz basic repetition rate. A part of the light was converted to other wavelengths with a fiber-optic-wavelength-converter (FOWC, SC-3.7-975, NKT, Denmark) through the dispersive wave and fiber-Raman gain effects. The wavelength of the output light could thus be modified by tuning the power of the laser directed to the FOWC. This design provided a new approach to multi-wavelength and fast-modulated light sources, with the available modulation frequencies from 54.77 MHz to several hundreds of GHz. However, the size of the light source was relatively large.

**Fig. 2.**
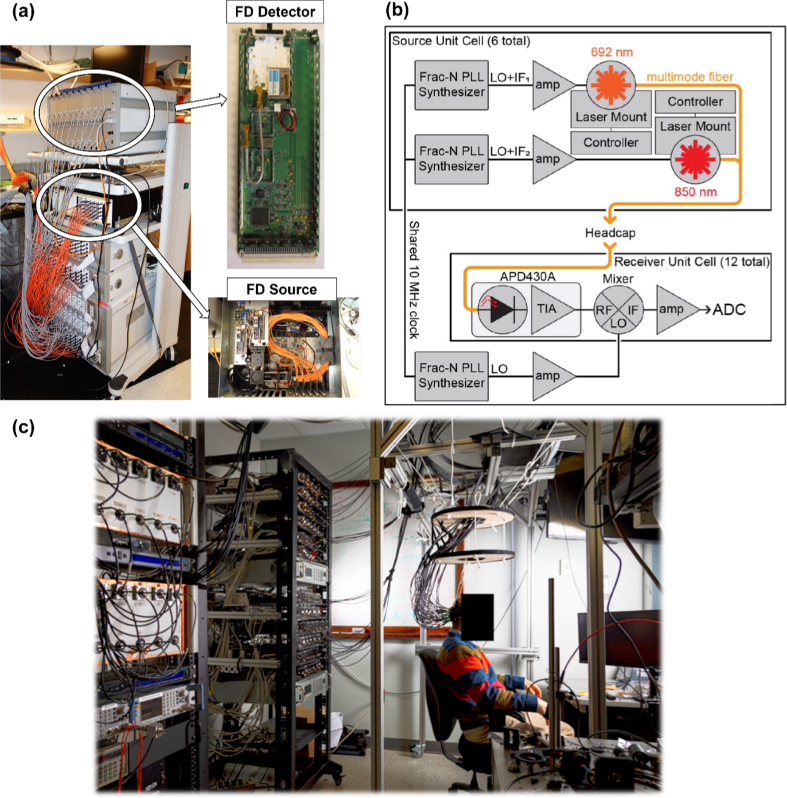
Some other examples of benchtop-based FD-NIRS systems. (a) The hybrid CW/FD-NIRS breast image system described by Zimmermann et al. The subfigure on the right demonstrates the FD-NIRS detector (upper) and source (bottom) components integrated into the system. This figure is taken with permission from [33]. (b) The schematic of the custom FD-fNIRS system described by Thompson et al. This figure is taken with permission from [34]. (c) The Dual Wavelength FD-fNIRS system described by Scholl et al., providing 1024 theoretical channels. This figure is taken with permission from [35].

In 2016, the idea of using FPGAs or system on a chip (SOCs) for NIRS signal processing was also adopted by Zimmermann et al. [33]; a full-sampling direct analogue-to-digital conversion FD-NIRS imaging system was presented, which implemented an FPGA for signal demodulation (see Fig. 2(a)). For the source input, a crystal oscillator (Connor-Winfield D75J, US) was utilized to generate the 50-MHz reference signal for the whole system. Two 50-mW laser diodes at wavelengths 685 and 830 nm (HL8338MG, Opnext, US) were implemented as light sources and the output light was switched to 24 different positions. A modular design was implemented for detection, in which twenty APD modules (C5331-04, Hamamatsu, Japan) were built on individually printed circuit boards (PCBs). The output signal from APD was then amplified, filtered, and converted into a differential signal. A high-speed ADC (LT2209, Linear Technology, US) sampled the signal (180 megasamples per second (MSPS)) with 16-bit resolution. The digitized signal was transmitted to an FPGA (Spartan-6 LX9, Xilinx, US) for signal demodulation. The demodulated signal was then collected by a microcontroller and sent to the PC.

This system [33] provided a total of 480 measurement channels at each wavelength. It achieved a high temporal resolution while maintaining a NEP below $1.4pW/\sqrt{Hz}$ and a dynamic range over 115 dB with 60 dB SNR at a 90-Hz bandwidth. Furthermore, the modular detection design provides the potential for expanded to higher channel numbers.

In 2021, Thompson et al. [34] proposed a FD-fNIRS system consisting of light sources at two wavelengths (692 and 805 nm) and twelve detectors were arranged in a head-cap, providing 72 channels at each wavelength (see Fig. 2(b)). Each of the light sources was modulated at an individual frequency around 211 MHz via a fractional-N phase-locked-loop (PLL), with an average optical power of 12.5 mW. The scattered light was detected by 12 APDs (APD430A, Thorlabs, US) and then down-converted, amplified, and digitized through a commercial audio digitizer. This work successfully implemented a FD-NIRS system for applications to the brain, providing extra decoding advantages which could be further employed when decoding more complicated neural activity patterns [34].

In the same year, Scholl et al. [35] presented a second-generation version of their dual-wavelength FD-fNIRS system. On the source side, 64 TO-can laser diodes were implemented with 32 operating at 690 nm (HL6738MG, Thorlabs, US) and 32 operating at 852 nm (L852P50, Thorlabs, US). The system used 64 custom-designed PCBs with PLL to modulate the laser diode light sources. Each board contained a PLL (ADF4351, Analog Devices, US) for modulation of signal generation. Thirty-two APDs (APD430A, Thorlabs, US) were used to detect the collected light, providing a total of 1024 theoretical channels at each wavelength. The electrical signal from each APD was then down-converted, amplified, balanced, and eventually digitized for further processing on a PC. The overall system structure is presented in Fig. 2(c). In 2021, the “Gen-3” system replaced the APDs of the system with silicon photomultipliers (SiPMs) to achieve a higher sensitivity and dynamic range [36]. An extra modulation board was also added to generate 64 TTL (transistor–transistor logic) waveforms thus providing a time-encoding operation. Time-encoding of the optical sources was employed to reduce shot noise. Moreover, all components of the Gen-3 system incorporated careful ground strategies, filtered power supplies, and electromagnetic shielding to decrease the signal contamination. In this Gen-3 system [36], optical sensitivity was indeed improved by implementing SiPM detectors, with an average NEP of 20.5 $fW/\sqrt{Hz}$ , and an SDS ranging from 13 to 40 mm was demonstrated. The system even had several valid channels at 70 mm SDS. Despite these many advantages, the system structure was relatively complex, and the footprint of the system was extremely large.

In addition to the numerous hardware developments of benchtop FD-NIRs devices over the last 20 years, a number of new algorithmic and analysis approaches have also been used to accelerate data processing. Some recent examples are briefly provided here. In 2020, Yoo et al. [37] demonstrated a deep learning method based on the Lippman-Schwinger Equation, which was capable of learning the non-linear photon scattering physics of the inverse scattering problem so as to calculate optical properties. This method can solve the non-linear inverse problem of DOT in an end-to-end manner and process new data within a few hundred milliseconds. This approach was demonstrated and validated previously using a method proposed by Heo et al. in the same group in 2018 [38]. In 2022, Kim et al. [39] demonstrated and validated a method based on the microscopic Beer-Lambert Law (BLL) to calculate the absorption coefficient changes of a sample from FD-NIRS data. This method claimed a 21.3 times faster speed than a traditional nonlinear least-squares fitting method. These methods achieved a shorter computation time in the FD-NIRS system, which provided the possibility to realize real-time FD-NIRS measurements with a higher frame rate and/or a greater number of channels. However, both algorithms were achieved by PC instead of an integrated module within the system itself.

### 2.3 Portable/wearable technologies

While benchtop technologies have been applied in several clinical studies [72–74], they have the common disadvantage of being somewhat cumbersome. This disadvantage significantly limits the use of FD-NIRS devices outside of laboratory environments, and can also hamper commercialization and clinical translation. In this section, we present recent progress toward portable and/or wearable FD-NIRS devices.

**Fig. 3.**
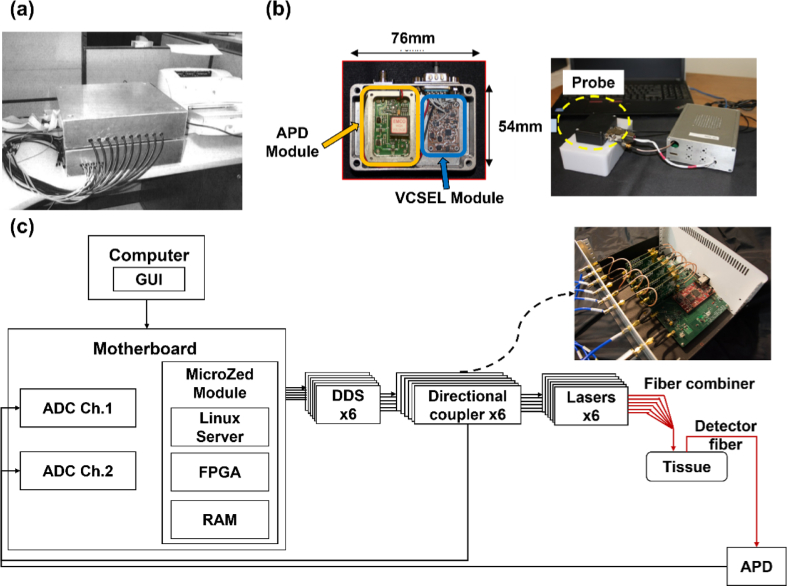
Examples of some portable/wearable FD-NIRS systems. (a) The portable FD-NIRS system for breast cancer detection described by Chen et al. This figure is taken with permission from [40]. (b) The FDPM instrument described by O’Sullivan et al. The subfigure on the left demonstrates the integrated probe containing a three-wavelength VCSEL package and a 3-mm diameter circular APD. This figure is taken with permission from [42]. (c) The schematic of the digital FD-DOS described by Torjesen, Istfan and Roblyer. The subfigure on the right demonstrates the core electronics of the system. This figure is taken with permission from [46].

In 2004, Chen et al. [40] presented a portable FD-NIRS system for breast cancer detection, as shown in Fig. 3(a). In this system, a 140 MHz sinewave signal was generated by a 10-dBm oscillator for source modulation. Three laser diodes (660, 780, and 830 nm) were utilized as light sources. The light from the sources was switched between 9 source positions by a high-speed optical switch (Piezosystem Jena, Germany). Ten identical acquisition channels for light detection were sealed in 10 small aluminum boxes for RF shielding. In each channel, an APD (S3884, Hamamatsu, Japan) was employed to detect the collected light. As a result, up to 90 measurement channels could potentially be produced at each wavelength. The measured signal was then down-converted, filtered and amplified. All the outputs from the detectors along with the reference signal were digitized with two DAQ cards (PCI-6070E, National Instruments, US), and then transmitted to a PC for processing. This system demonstrated a dynamic range of approximately 85 dB. Moreover, the utilization of a high-speed optical switch (∼2 ms switching time) facilitated a reasonable imaging acquisition speed of 0.5 frames per second. In addition, RF shielding of the detectors was effective to improve noise isolation. However, this system is not without its limits. Firstly, the total weight of the system was ∼12 kg. Though it was significantly lighter than most benchtop devices, is still heavy for a “portable” device. Moreover, the SDS of the system was unclear, as was the effective channel count provided by the device in practice. Furthermore, there were no values of latency provided for this system. It is therefore difficult to fully assess the claim of near real-time data acquisition.

There have been several works aiming to improve and miniaturize FD-NIRS technologies by utilizing new optical components and/or designing custom onboard systems. In 2008, No et al. [41] proposed a FD-NIRS system for breast scanning. In this system, a PLL was employed to generate modulation signals. Four laser diodes at the wavelengths of 681, 783, 823 and 850 nm were utilized as light sources, with an average output power of 20 mW. A handheld probe was implemented which included source optical fibers and an APD. The APD detected the remitted light and output an RF signal. The signal was then filtered, down-converted (to lower frequency) and amplified. A phase detector chip was implemented to measure the power and phase shift of the down-converted signal. The signal was then digitized by a built-in ADC and transmitted to a PC for further processing. In 2017, O’Sullivan et al. [42] modified the handheld probe by integrating three commercial non-tunable Vertical-Cavity Surface-Emitting Laser (VCSEL) diodes in a 5.6 mm package (Vixar, US) along with a 3 × 3 mm circular active area APD (S6045-05, Hamamatsu, Japan). A single measurement channel at each wavelength with an SDS of 20 mm was generated. The integrated probe and the system are shown in Fig. 3(b). In 2018, a tunable VCSEL was designed at the same research group by Kitsmiller et al. [43] for FD-NIRS systems.

These publications demonstrated some encouraging advancements in the development of a wearable FD-NIRS system. The system developed by No et al. [41] achieved a measurement speed of <200 ms per wavelength with a noise level of -88 dBm and a dynamic range of 100 dB. The modified system with VCSEL [42] achieved FD measurements at a large frequency range from 50 to 500 MHz. It also achieved an optical power of 11.6 mW and a ratio of power consumption to the average optical power of 13.4 [42]. These works [42,43] demonstrated the feasibility of using VCSELs in wearable FD-NIRS systems. On the other hand, there were still some drawbacks in the system. The imaging capability was limited due to the inadequate channel number. In addition, the relatively low output power of the VCSELs utilized in this work may lead to relatively low SNR and consequently limit signal quality, particularly for deeper tissue regions.

In 2014, Roblyer et al. [44] demonstrated a digital FD-NIRS system. The RF modulation signal from direct digital synthesizer (DDS) was amplified, split, and directed to an RF switch and an ADC separately. The RF switch selected the light from six laser diodes (656, 687, 778, 814, 824, 852 nm), each with an output power of 20 mW. The light was then directed to the subject’s tissue through a six-in-one fiber. The scattered light was detected by an APD (S6045-05, Hamamatsu, Japan), then filtered and sampled by a 12-bit dual-channel ADC (1.8 gigasample per second (GSPS) per channel) and compared to the reference RF signal. In 2014, Jung et al. [45] utilized digital under-sampling to reduce the complexity, power consumption, and cost of the system. The modified system utilized a Lattice ECP3 field-programmable gate array (FPGA) to process the signal from APD, including correcting the phase of the under-sampled signal. In 2017, several parts of this digital FD-NIRS system [46] were integrated into a single motherboard (ZedBoard MicroZed Zynq-7010 SOC, Xilinx, US) which can generate and measure RF signals while communicating with the host PC (see Fig. 3(c)). Six DDS boards were used to generate the modulation signals. In 2021, Applegate et al. [47] proposed a high-speed look-up table (LUT) approach for optical properties estimation. In the same year, Istfan et al. [48] adapted the system for sternocleidomastoid (SCM) measurement of oxygen saturations with only two laser diodes at 730 and 850 nm (FMXL730-030YFGA, Blue Sky Research, US, and LP852-SF30, Thorlabs, US).

These developments [44–46,48] successfully implemented the direct digital sampling of the modulated signal in FD-NIRS systems. The system in [46] provided 6 optical channels (with 6 different wavelengths) at an SDS ranging from 10 mm to 30 mm. It achieved an SNR of 46.3 dB while the accuracies of the absorption coefficient and the reduced scattering coefficient measurements were quoted as 5.3% and 5.5% respectively. The integration of the RF detection and measurement module improved the acquisition speed and reduced the system size and cost. Moreover, the LUT method proposed by Applegate et al. [47] could potentially be integrated into the FPGA module, thus speeding up the signal processing. On the other hand, there were also some improvements to be made to the system. For instance, the system applied individual DDS board for the modulation of each laser diode, which increased the cost as well as the size of the system. Besides this, the size of the integrated motherboard could be further reduced by utilizing a more compact FPGA board such as Zynq UltraScale + MPSoC [76] or Snickerdoodle [77]. Furthermore, the channel number of the system was limited.

**Fig. 4.**
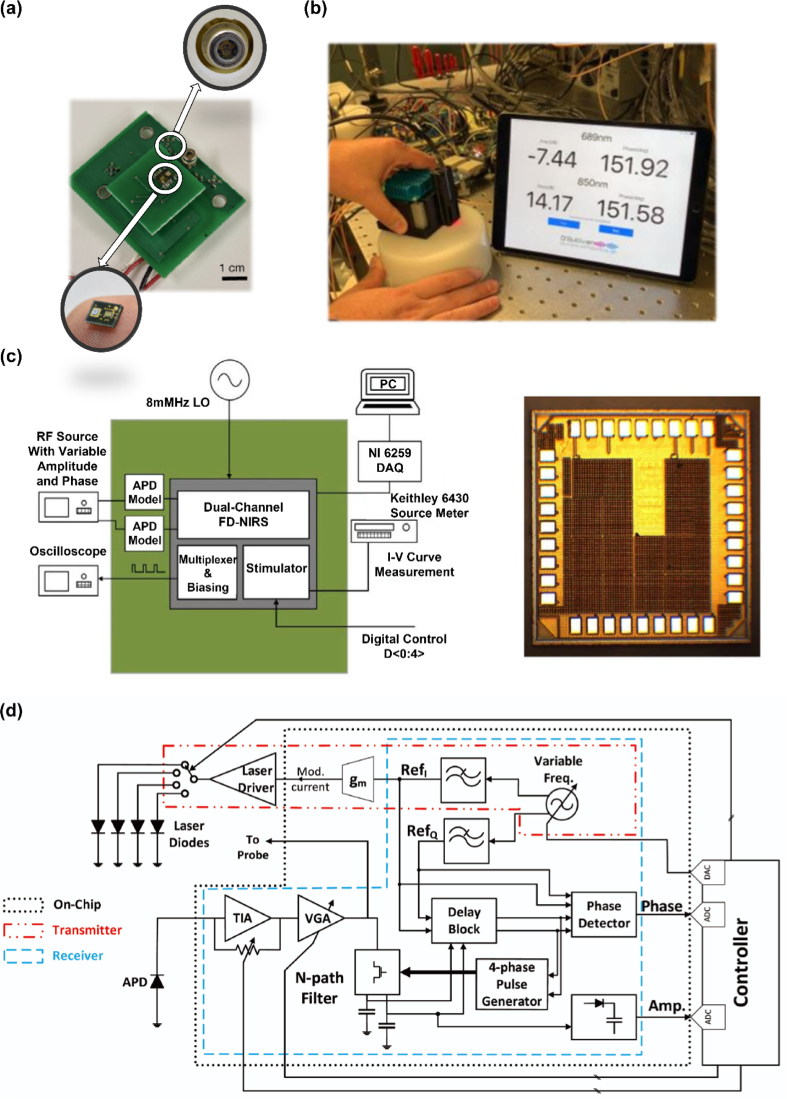
Some recent examples of portable/wearable FD-NIRS systems. (a) The miniOptode integrated with the VCSEL source (top) and APD detector (bottom) for the FD-NIRS system described by Istfan et al. This figure is taken with permission from [50]. (b) The photograph of the handheld FD-NIRS system described by Stillwell et al. This figure is taken with permission from [58]. (c) The schematic and the chip micrograph of the CMOS-based system described by Miao and Koomson. This figure is taken with permission from [62]. (d) The schematic of the CMOS-based system described by Yazdi. This figure is taken with permission from [65].

In 2018, Zhao et al. [49] proposed a pulse oximeter with the capability of quantifying and displaying the absolute concentration of oxy- and deoxy-hemoglobin in real time. The system was developed based on the digital NIRS system described in [46] above. Five DDS boards were employed to modulate the light, and the scattered light was detected by a 3 mm active area APD (S11519-30, Hamamatsu, Japan) with an SDS of 10 mm. The output of the APD was consequently digitized by an ADC and compared to the reference signal on a SOC where the amplitude and phase of the light were calculated. In 2019, a miniaturized single source-detector optode (miniOptode) that integrated a multi-wavelength (660, 680, 775 and 795 nm) VCSEL (Vixar, US) and a small format APD (S13282-01CR, Hamamatsu, Japan) was presented [50]. The miniOptode is shown in Fig. 4(a). In 2021, the system in [46] was further improved via the utilization of the programmable logic component of the SOC [51]. Six measurement channels at six different wavelengths with an SDS of 20 mm were provided. By utilizing a bank of 12 Goertzel filters in the programmable logic component, the upgraded system can directly calculate the amplitude and phase values from each channel at each modulation frequency, providing increased data processing speed. Moreover, a fast LUT method (originally proposed in [47]) was implemented by the programmable logic component to recover optical properties based on the signal amplitude and phase calculated by the SOC.

Compared to the benchtop system [46], the modified system [51], provided an increased measurement speed and reduction in data load of about two orders of magnitude. Besides this, the implementation of the compact VCSELs in miniOptode [50] greatly decreased the footprint while maintaining the accuracy of the optical properties estimation. The miniOptode [50] achieved an SNR of 53.5 dB and a dynamic range of 54.4 dB at 13 mm SDS. It also provided a valid bandwidth spanning at least 50 to 249 MHz with its active area 25 times smaller than the 1 mm detectors commonly used for FD-NIRS. These works demonstrated promising results in developing a wearable FD-NIRS device. However, the SNR of the miniOptode [50] would decrease sharply at longer SDS, which limited its capability to monitor deeper tissues such as the brain. Furthermore, the system has yet to be scaled to the channel numbers required for tomographic reconstruction and is thus unable to recover 3D information.

In the above work [50], VCSELs were used as the light source to reduce the size of the system. In a similar manner, smaller detectors have also been investigated to minimize system size. In 2019, Wang et al. [52] verified the feasibility of using SiPMs as the detectors for FD-NIRS/DOT. In the same year, Kitsmiller and O’Sullivan [53] replaced the APD with SiPM in a FD-NIRS system, leading to a 5-30 dB increase in SNR. The SiPM was then implemented to realize a lightweight, handheld, real-time FD-NIRS system in the same year [54]. A SOC platform was utilized to control and process the signals of the system, including the DDS (AD9912, Analog Devices, US) used for signal modulation. For detection, a small-scale PCB integrated with SiPM was constructed. The output from the SiPM was then digitized by an ADC (AD9613, Analog Devices, US) and compared with the reference RF signal from the DDC module. The ADC then streamed the data to the FPGA on the SOC platform for optical properties calculation. In 2020, Kitsmiller et al. [55] explored SiPM characteristics to improve FD-NIRS system performance. In the same year [56], the performance of SiPMs in FD-NIRS was evaluated and demonstrated several advantages over APDs, including higher sensitivity, larger dynamic range, higher SNR, and smaller size; but also exhibited some limitations, including overall bandwidth. Another advance occurred in 2020, when five edge-emitting laser diodes at 690, 785, 808, 830 and 805 nm and a VCSEL at 940 nm were integrated into a PCB as the light source [57]. In 2021, the calculation speed for optical properties was further enhanced by implementing a k-nearest neighbor (KNN) LUT on the SOC platform [58], resulting in 13 - 322 times improvement (in SOC) and 170 - 2,200 times improvement (in a modern CPU, AMD 3960X, 3.8 GHz) when compared to similar work [47]. The photograph of the handheld system [58] is shown in Fig. 4(b).

The system [57,58] provided a single optical channel with an SDS of 15-30 mm at each wavelength. It also achieved a noise floor of -68 dBm at an SDS of 23 mm. Besides this, VCSEL and SiPM were employed as sources and detectors which reduced the size of FD-NIRS systems. Furthermore, the implementation of a SOC platform with FPGA for signal processing also increased the portability of the system.

The works above embedded the system on a SOC platform to achieve miniaturization of low-channel-count devices. Alternative approaches based on CMOS-integrated solutions have also been proposed. In 2011, Yun et al. [59] proposed the designs of CMOS-integrated APDs and frequency-mixing transimpedance amplifiers (TIA, converting current from APD to output voltage). The TIA can realize simultaneous down-conversion and amplification of the photodiode current. In 2013, Sthalekar and Koomson [60] utilized the TIA architecture from [59] and proposed a 4-channel sensor design on a 2.25 mm^2^ chip for FD-NIRS measurements. The 100 MHz current signals from the photodetector were converted to a high-frequency voltage with the TIA. The voltage signal was further down-converted by a heterodyne Gilbert cell mixer and an external oscillator. An integrated time-to-digital converter was then employed to perform phase and amplitude measurements. In 2017, the MOSFET (Metal Oxide Silicon Field Effect Transistor)-based TIA was replaced with SiliconGermanium (SiGe) heterojunction-bipolar-transistor based TIA [61]. The modified chip [61] provided lower noise and higher bandwidth detection. In the same year, the chip [61] was integrated with transcranial direct current stimulation (tDCS) to achieve simultaneous brain stimulation and monitoring [62]. In 2018, the performance of the practical measurement at 685 and 853 nm was evaluated [63]. In 2022, a design integrated with laser diodes and DDS was presented [64]. This design could potentially be combined with the chip in [60] to miniaturize the FD-NIRS system. The schematic and the chip micrograph of the system [62] are shown in Fig. 4(c).

These works [59–62] integrated a single-channel FD-NIRS measurement system on a 2.25 $mm^{2}$ chip, with a dynamic range of 33 dB. It also reached a phase resolution of ${\mathrm{0.2}}^{\circ }$ with a power consumption of 30 mW [62]. The low-power integrated design provided the potential for construction of a wearable FD-NIRS device. There were, however, some disadvantages with these systems [59–62] to note. The SNR of the system was limited when the photocurrent dropped below 10 nA, thus affecting the phase measurement resolution. This limitation would be a concern when integrating a low-power light source into the system. The scalability of these systems also remains unknown.

In 2017, Yazdi [65] proposed a fully integrated FD-NIRS system in 180 nm standard CMOS. An external controller was applied to tune the control voltage applied to a quadrature oscillator on the chip, thus adjusting the modulation signal frequency generated by an oscillator. The modulation signal was then filtered and directed to modulate the external laser diodes. The light was first detected by an external APD, then amplified by an integrated TIA and variable gain amplifier (VGA), and further filtered by an N-path filter. The phase and amplitude of the filtered signal were subsequently calculated by the N-path filter-based phase measurement unit and a pseudo-logarithmic, amplifier-based amplitude measurement unit. The schematic of the system is illustrated in Fig. 4(d).

This integrated system [65] provided a potential solution for low-power, low-cost wearable FD-NIRS devices. The chip provided a single channel measurement with an SDS of 20 mm at each wavelength. It achieved a sensitivity of 70 $nA_{rms}$ and phase and amplitude measurement errors of $2^{\circ }rms$ and 50 $nA_{rms}$ at 400 MHz modulation frequency. Nevertheless, the relatively low output power of the utilized VCSELs would lead to low SNR and thus limit SDS and thus sensitivity to deeper tissues. In addition, external light sources and detectors, as well as the controller, were also required in this system [65]. These drawbacks would be a concern when implementing the system in a truly wearable form factor.

Several works have aimed to improve wearable FD-NIRS system performance through system component integration and new signal processing schemes. In 2022, Chen et al. [66] presented a FD-fNIRS measurement integrated circuit (IC). A mixer-first analog front-end (AFE) on IC was utilized for demodulation to reduce the system power, while an on-chip Σ−Δ phase-to-digital converter (PDC) was implemented to lower the laser modulation frequency while achieving a phase resolution of $0.0096^{\circ }$ . Koh and Bae presented a FD-NIRS IC which integrated a laser driver into the chip in 2022 [67]. In the same year, Scammon et al. [68] proposed to use frequency-division multiplexing scheme to differentiate wavelength using a single detector, and also reduce the system by removing the optical switch. These works provided various approaches to reduce the size and energy consumption to enable a wearable FD-NIRS system.

There are also some commercial products that have the potential to be integrated into FD-NIRS systems. For example, the SR4000/SR4500 Time-of-Flight system (Mesa Imaging, Switzerland) [69] can modulate its illumination LEDs, while also providing a CCD (charge-coupled device)/CMOS (complementary metal-oxide-semiconductor) sensor for phase measurement of the modulated light. The size of the SR4000 is 65 **×** 65 **×** 68 mm^3^, with a weight of 470 g, which may hold the potential to be utilized in portable FD-NIRS system in the future.

## 3. Light sources and detectors, and phase measurement schemes

The focus of this section is primarily to summarize the types of light sources and detectors, as well as the phase measurement schemes, that are currently employed in FD-NIRS systems [14,17–69]. Note, key characteristics of these light sources and detectors, and phase measurement schemes are summarized in Table S1 and S2 respectively as Supplement 1.

### 3.1 Light sources

In many existing FD-NIRS systems [19–21,23,25,27,29,30,32,34,35,39–41,44–48,51,52,54,56–58,60–62,65], Edge-Emitting Lasers (EELs) have been widely applied as light sources. In an EEL, light is emitted from the edge of the substrate, and an EEL offers relatively high power density and wall-plug efficiency, and good beam uniformity [78].

In recent years, VCSELs [42,43,50,57,58] has become an alternative option to act as light sources in NIRS systems. In a VCSEL, the light is emitted perpendicular to the mounting surface, as opposed to an EEL, which emits light parallel to the surface. Compared to EEL, VCSEL holds several advantages, including 1) better wavelength variability, 2) higher beam quality, 3) lower power consumption, and 4) lower cost [79].

Of note, the source powers in all the discussed devices [19–21,23,25,27,29,30,32,34,35,39–48,50–52,54,56–58,60–62,65] are expected to meet the skin safety limits (i.e. maximum permissible exposure) suggested by IEC 60825 standard [80,81].

### 3.2 Light detectors

A photodiode (PD) is fundamentally a *p-n* junction operated under reverse bias, and in past years PDs have been widely utilized in CW-NIRS devices [82]. However, without internal gain, it is obviously challenging to apply PDs to detect light at microwatt to picowatt (or lower) levels. Therefore, PD becomes a typical factor that constrains the system sensitivity of FD-NIRS systems.

PMTs are an alternative to PDs to act as detectors for NIRS devices. PMTs provide ultra-high internal gains of 10^4^ - 10^9^ and ultra-low dark currents (∼0.3 pA at 25 °C [83]) which has the potential to provide exceptionally high system sensitivity. However, the devices are typically bulky, often require active cooling, require high bias voltages (∼ 1000 V) and are highly vulnerable to damage if exposed to excess light, which can limit their applicability.

In comparison, thanks to their relatively small size and in-built amplification, another type of photodetectors, APDs, have been widely employed in FD-NIRS devices [18–25,32–35,37–51,53,54,56–68]. In particular, APDs possess internal gains of 10^2^ - 10^3^ and low dark currents (∼0.1-100 nA at 25 °C [84]), providing exceptional SNRs. However, these also require high reverse bias voltage (∼ 200 - 500 V). For both PMT and APD, the high reverse bias voltages and consequently the large-size high-voltage converters required make them somewhat impractical to be utilized in wearable FD-NIRS systems.

In recent years, SiPMs have become a promising approach to act as optical detectors in FD-NIRS systems [36,52,53]. A typical SiPM consists of around 500 - 4000 single-photon avalanche diodes (SPADs) that operate in single-photon counting mode. SiPMs have demonstrated high internal gain (10^5^ - 10^7^), low dark currents, and excellent SNR while requiring only a relatively low reverse bias voltage (∼ 20 - 50 V). This significantly lower voltage permits the use of miniaturized high-voltage modules that can support the development of wearable FD-NIRS systems. On the other hand, the linearity in responsivity and the speed of frequency response of SiPMs (∼10 dB delay at 500 MHz) could be further improved relative to APD [53].

### 3.3 Phase measurement schemes

In Section 2 above, various methods have been implemented to perform phase measurements. A conventional approach is to use an external benchtop device such as a network analyzer, or lock-in amplifier. These devices, in which each component was primarily built with well-established, commercially available elements, do not tend to have challenging system size or operating voltage requirements. As a result, achieving high performance phase measurements (in terms of e.g. SNR, dynamic range etc.) is more straightforward with the smaller-footprint devices discussed in Section 2.3. These approaches are often used for benchtop devices [14,21] or for testing the performance of optoelectronics components [32,42,50,53,54,56].

Another common solution is to down-convert the signal by means of a heterodyne mixer and subsequently perform the measurement at lower frequencies. Some systems implement the mixing process by means of modules on an integrated board or chip [58,60–63,65], while others use external signal generators and mixing devices to down-convert the signal [27–29,31,34–36,40]. The down-converted signal is then used for phase calculation via FPGA or PC or DAQ. The use of chips with integrated modules for light detection and phase measurement seems the most promising solution for phase detection in a wearable form factor, provided that other components (e.g. light sources, signal processing components) can be reasonably matched and miniaturized. For the integration on the board (often with additional integrated amplifier components) or external devices for down-conversion, the solution is obviously larger in size than an integrated CMOS chip, but can achieve greater signal gain, better stability, and more freedom in signal reception and processing after the down-conversion is completed. This route also has the potential to be implemented in wearable devices.

There are also devices that sample directly via a high-speed ADC and subsequently use an FPGA or PC to calculate the phase change [33,39,44,46,48,49,51,57,58]. As this method is based on a fully digital approach, the data acquisition process is less likely to be influenced by environment electromagnetic interference, making it particularly appropriate for extra-laboratory application environments where EM noise is harder to control. With the guaranteed performance of the high-speed ADC and the data acquisition and processing capability of the FPGA/SOC, the phase measurement accuracy of the system can also be ensured, while also allowing for the target measured signal to be outputted to the host device. However, most research groups are currently designing with commercially available SOC platforms, so there is still room for optimization in terms of system size. Moreover, the power consumption and heat dissipation of FPGA devices will likely need to be addressed in wearable FD-NIRS device development.

Other options, such as using a low-speed ADC for sampling and subsequent phase correction of the signal with an FPGA [45], and using an I/Q demodulator to sample the signal and process it with a PC [25,37], have also been mentioned in this review. The use of FPGAs for the phase correction of under sampled signals may result in phase errors which may not be suitable for situations where accurate phase measurements are required. The use of an I/Q demodulator may reduce costs to some extent, but it still requires subsequent processing by an external device PC to obtain the phase values.

## 4. Current clinical applications using FD-NIRS

### 4.1 Search strategies for FD-NIRS applications to clinical care

The following clinical studies were identified using a Google Scholar and Web of Science search of the terms [(frequency-domain OR FD) AND (near-infrared spectroscopy (NIRS) OR fNIRS OR diffuse optical spectroscopy OR DOS)]. This search returned 94 results. The abstracts of all were reviewed to determine whether the study presented a clinical application of FD-NIRS since 2000. Of the 94 studies identified, 37 met these criteria, all of which are described here. This search was not intended to be comprehensive, but rather provide an in-depth review of the current state of FD-NIRS applications to clinical care. Note, almost all the clinical applications below utilized the commercially available FD-NIRS systems, such as OxiplexTS and Imagent systems (ISS Inc., Champaign, IL, US), and PMD system (NIM Incorporated (dissolved in 2009), Philadelphia, PA, US). We will discuss these systems in turn below.

### 4.2 OxiplesTS system in clinical applications

The OxiplesTS system, a 2-channel FD-NIRS system from ISS [85], has been widely applied to clinical neuromonitoring in adults, particularly for those at-risk of cerebrovascular-related brain injury. Using this system, Calderon-Arnulphi et al. (2007) investigated FD-NIRS as an intraoperative monitoring technique for adult patients undergoing brain surgery [86]. Of the 25 patients studied, 5 had clinical ischemic events during surgery, all of which corresponded with a decrease in oxy-hemoglobin (HbO) concentration, total hemoglobin (HbT) concentration, and oxygen saturation (SO2) [86]. Another study used the OxiplexTS system in combination with electroencephalography (EEG) in adult neurosurgical patients undergoing intubation. Increased HbO concentration, decreased deoxy-haemoglobin (HbR) concentration, and increased EEG response entropy were observed, suggestive of intubation-induced stress and autonomic nervous system activation [87]. Other applications to cerebral hemodynamic monitoring include: assessing the effect of administration of phenylephrine to anesthetized patients during hyper-, hypo-, and normocapnia conditions [88]; assessing dynamic cerebral autoregulation in neurocritical care patients [89]; and the assessing the effects of head-up-tilt and hyperventilation in propofol-remifentanil anesthetized non-neurosurgical patients [90]. The OxiplexTS system has also been used to demonstrate reduced microvascular cerebral oxygenation in patients with multiple sclerosis [91] and reduced regional cerebral oxygen saturation in patients with stroke the first few hours after onset [92].

The OxiplesTS system has also been extensively applied in cerebral haemodynamic monitoring in neonates. Roche-Labarbe et al. (2010) combined FD-NIRS with DCS to investigate the validity of applying stable adult values of the CBV-CBF (Cerebral blood volume-Cerebral blood flow) relationship to preterm neonates [93]. This group further applied this system to investigate FD-NIRS/DCS as a perioperative monitoring technique for infants with congenital heart disease at risk of brain injury [94,95]. The FD-NIRS/DCS system was also demonstrated to be suitable as a bed-side monitoring technique for premature newborn infants [96], for premature newborn infants with low-grade germinal matrix intraventricular hemorrhage, for newborn infants with hypoxic ischemic encephalopathy [97], and for infants undergoing hypothermic cardiopulmonary bypass during surgery [98]. The system was also used to compare regional hemispheric differences in hemodynamics between term-born and preterm-born infants [99,100], as well as sexual dimorphism between male and female healthy newborn infants [101].

Demel et al. (2015) combined the OxiplexTS FD-NIRS system with Doppler sonography to assess cerebral perfusion during the first three days after birth in term-born and preterm infants [102]. This group also used FD-NIRS to demonstrate that brain-water content can undergo maturational changes during the prenatal period, which can lead to erroneous deviations in SO2 readings as measured by traditional CW-NIRS unless otherwise accounted for [103].

Further, Tian et al. (2017) used the OxiplexTS system to assess cerebral oxygenation in pediatric patients undergoing extracorporeal membrane oxygenation (ECMO) [104]. Regional abnormalities in oxygenation were identified in ECMO patients as compared to a cohort of healthy controls.

### 4.3 Imagent system in clinical applications

Similar to the OxiplesTS system, the Imagent system has found applications in cerebral monitoring in adult, neonatal and pediatric populations. Zhang et al. (2021) employed the Imagent system to study cerebral hemodynamics in adult patients with obstructive sleep apnea [72]. In this study, longer apnea events were associated with stronger cerebral desaturation, and specific brain states (i.e. sleep states, arousal) were found to differentially affect cerebral hemodynamics.

In pediatric populations, Franceschini et al. (2007) used the ISS Imagent system to demonstrate the feasibility of multi-wavelength, multi-distance FD-NIRS for bedside monitoring of cerebral hemodynamics in healthy infants [73]. The entire system could be fitted on a trolley such that it could be wheeled in and out of the patients’ rooms. Results demonstrated that the system could be used to detect highly significant and regionally specific changes in cerebral hemodynamics. In a follow-up study, the system was applied to determine whether FD-NIRS measurements could distinguish between neonates with and without brain injury [74]. Cerebral blood volume and the cerebral metabolic rate of oxygen were found to be significantly higher in brain injured neonates as compared to infants without injury. Lee et al. (2022) used a combined FD-NIRS/DCS system to assess cerebral hemometabolic stress in pediatric sickle cell anemia patients following blood transfusion [105]. Pre- and post-transfusion values of oxygen extraction fraction (OEF), CBV, and CBF were compared, and expected decreases across all parameters were observed post-transfusion.

The Imagent system has also been applied to study the hemodynamics of the female cervix during pregnancy as a potential predictor of preterm labor. Preterm labor can lead to significant risks for infants, and identification of women at risk can allow for early intervention to delay delivery and improve infant outcomes [106]. Baños et al. (2007) used a modified Imagent system to investigate the changes in optical properties of the cervix of pregnant women during drug-induced cervical ripening. For this study, a probe was also designed to allow for positioning of the optical fibers directly on the cervix. A decrease in HbT and HbR and an increase in total water content were observed, suggestive of tissue edema during cervical ripening [107]. Two follow-up studies were performed by this group using this system, one of which was a longitudinal study assessing the changes in optical properties across gestation [106], while the other assessed these changes across phases of the menstrual cycle [108]. Altogether, the results of these studies supported FD-NIRS as a diagnostic tool for assessing cervical health and cervical ripening as a predictor of preterm labor. Relatedly, Stahel et al. (2009) used a modified Imagent system to assess changes in the optical properties of breast tissue during birth-control mediated menstrual cycles as compared to spontaneous (natural) cycles [109]. The optical properties of tissue were found to demonstrate a biphasic pattern across menstruation in subjects with spontaneous cycles as opposed to those on birth control.

### 4.4 Other examples of systems used in clinical applications

Watzman et al. (2000) applied a ‘prototype’ FD-NIRS system (PMD, NIM Incorporated, Philadelphia, PA, US) to study cerebral hemodynamics during cardiac catheterization in pediatric patients with congenital heart disease [110].

Zavryiev et al. (2021) employed a combined FD-NIRS and DCS system to monitor cerebral hemodynamics in adult patients during hypothermic circulatory arrest [111]. The advantage of using FD-NIRS and DCS in combination is that it allows for measurement of oxygen saturation (from FD-NIRS and cerebral blood flow index) and CBF (from DCS), as well as cerebral metabolic rate of oxygen (CMRO2), which is considered a more comprehensive measure of brain health [29]. The FD-NIRS/DCS system was applied to study adult patients undergoing cardiac surgery and requiring hypothermic circulatory arrest. Changes in CBF, oxygen saturation, and cerebral metabolic rate of oxygen were detected in response to hypothermic circulatory arrest intervention, and the authors concluded that this system could be used to inform cerebral protection strategies during hypothermic circulatory arrest.

## 5. Discussion and conclusion

### 5.1 Summary of current status of FD-NIRS technologies

FD-NIRS is highly advantageous in that it allows for direct quantifications of the scattering and absorption coefficients of sampled tissues and, consequently, the chromophore concentrations of interest. FD-NIRS has been increasingly applied across varied clinical applications as described in this article. In the evolution of this field, the development of miniaturized, high-density FD-NIRS technology is a critical next step.

Our review presents the state-of-art in FD-NIRS technologies. To date, several benchtop systems have been successfully developed [14,17–39], and some of them [17,23–25,27,33,35] have achieved a relatively large channel counts. Particularly, the systems in [19,20,22–28,31,37,38] have demonstrated the capability to produce 2D images. Despite these successes, these technologies are subject to the following limitations: (1) limited portability: large size, heavy weight, and fixed location; (2) relatively poor ergonomics; and (3) a limited capability to produce images. The work in [23–25] incorporated a multi-channel optical switch with “shared” light sources so as to multiply the number of measurement channels, while the systems in [27,33,35] utilized individual light sources, detectors and control/driving electronics to produce relatively large channel counts; however, both methods led to large device size and cumbersome cabling, as shown in Fig. 2 above. Other systems only provide limited channel numbers (less than 50), while remaining relatively large in size (see Fig. 1). Moreover, the imaging capability has not been validated by most of the available systems: only 13 (of 54 in total) systems in [20,24–28,31,37,38,40,46,51,58] demonstrated the functionality of 2D imaging, and only the systems in [22,23] accomplished 3D images to any degree.

To overcome some of these limitations, several attempts at constructing portable/wearable FD-NIRS devices have been made [40–68]. While encouraging, there remain some concerns with these systems. Almost all the portable/wearable systems could only provide a limited channel number (less than 10), which then produced limited information and zero capability to generate images. The portable system developed in [40] can provide 90 measurement channels with the help of an optical switch; however, it was still relatively large (∼12 Kg). There is an emerging trend to develop portable/wearable FD-NIRS devices via CMOS microchips [59–67], which potentially holds several advantages, including a small footprint, low power consumption, and potentially improved measurement accuracy with reduced noise. However, the channel numbers of these systems remain limited to date, and most exhibit constrained SDS and dynamic range.

Improvements in the key components of light sources, light detectors, and phase measurement units will be vital factors for the constructions of new FD-NIRS technologies. It can be seen from Sections 3.1 and 3.2 that VCSELs and SiPMs have the potential to be the optimal choice for light sources and detectors, respectively. However, as emerging technologies, pre-packaged VCSELs and SiPMs with appropriate wavelengths, optical power, and package size remain relatively rare. In terms of phase measurement units, FPGA-based and microchip-based solutions are encouraging. Their potential advantages include a small footprint, low-power consumption, high speed, and high accuracy. Despite this, implementing these approaches is expensive, labor-intensive, and time-consuming.

For FD-NIRS systems to take a greater foothold in clinical spaces, devices would ideally be smaller, more portable, low-cost, and user-friendly for clinical staff. Better ergonomics is a particular concern for using these systems in vulnerable populations, such as patients in intensive care or infant patients. Presenting data in real time in formats that are clinically interpretable is also a key consideration. Additionally, the ease of integration with other neuromonitoring technologies, such as DCS or EEG, would allow for a more complete picture of brain health. Finally, achieving high density systems would allow for the expansion to 3D imaging, and therefore applications beyond single-site oxygen and metabolism monitoring.

### 5.2 Potential future directions and improvements for FD-NIRS technologies

Looking to the future of FD-NIRS systems, improvements in imaging resolution, high-speed ADCs, sensitive front-end optoelectronics and amplifiers, accurate phase detection, and precise logic control will be necessary to obtain the large dynamic range and precise measurement needed for large channel count FD-NIRS systems. Achieving the hardware improvements that allow for improved device ergonomics (i.e. to move towards devices that are miniaturized, lightweight, flexible, comfortable) will continue to remain a key goal. More suitable phase measurement schemes and data interfaces will be required to ensure a large channel number, large dynamic range, and high-speed data acquisition and transmission are possible. Additionally, VCSELs and SiPMs with compact size, appropriate wavelengths, and low prices will likely be the preferred choice for developing the next-generation, of miniaturized, wearable, and high-density FD-NIRS systems. To achieve these goals, extensive effort is needed in RF design, optoelectronic integration, mechanical design, signal processing, and fabrication.

### 5.3 Conclusion

Within the next five years, with a multidisciplinary effort from engineers, computer scientists, and clinicians, it seems likely that miniaturized, wearable, fibreless, high-density, FD-NIRS/DOT will become readily available. The advent of these technologies will dramatically accelerate the growth of FD-NIRS/DOT throughout neuroscience and clinical neurology, and will also have wide-reaching implications for sectors including brain-computer interfacing, human-robot interaction, rehabilitation, sports monitoring, and personalized healthcare.

## Data Availability

No data were generated or analyzed in the presented research.

## Supplemental document

See Supplement 1 for supporting content.
