# Supplementary material for: Review of recent advances in frequency-domain near-infrared spectroscopy technologies [Invited]
Source: Biomed Opt Express. 2023 Jun 12;14(7):3234–58. doi: 10.1364/BOE.484044 (PMC10368025; doi:10.1364/BOE.484044)
Supplement: Supplementary file 1 [file boe-14-7-3234-s001.pdf]

Supplemental Document

Biomedical Optics EXPRESS

Review of recent advances in frequency-domain near-infrared spectroscopy technologies [Invited]: supplement

XINKAI ZHOU,[1,†] YUNJIA XIA,[1,2,†] JULIE UCHITEL,[3,†] LIAM COLLINS-JONES,[2] SHUFAN YANG,[1,4] RUI LOUREIRO,[5] ROBERT J. COOPER,[2] AND HUBIN ZHAO[1,2,*]

[1] *HUB of Intelligent Neuro-engineering (HUBIN), Aspire CREATe, IOMS, Division of Surgery and Interventional Science, University College London (UCL), London, HA7 4LP, UK*
[2] *DOT-HUB, Department of Medical Physics & Biomedical Engineering, UCL, London, WC1E 6BT, UK*
[3] *Department of Paediatrics, University of Cambridge, Cambridge, CB2 0QQ, UK*
[4] *School of Computing, Engineering & Build Environment, Edinburgh Napier University, Edinburgh, UK*
[5] *Aspire CREATe, Department of Orthopaedics & Musculoskeletal Science, UCL, London, HA7 4LP, UK*
[†] *These authors contributed equally to this work.*
[*] *hubin.zhao@ucl.ac.uk*

This supplement published with Optica Publishing Group on 12 June 2023 by The Authors under the terms of the Creative Commons Attribution 4.0 License in the format provided by the authors and unedited. Further distribution of this work must maintain attribution to the author(s) and the published article's title, journal citation, and DOI.

Supplement DOI: https://doi.org/10.6084/m9.figshare.23274446

Parent Article DOI: https://doi.org/10.1364/BOE.484044

Supplementary Document to -

Review of recent advances in frequency-domain near-infrared spectroscopy technologies

List of abbreviations

**AFE**	analog front-end
**APD**	avalanche photodiode
**BLL**	beer-lambert law
**CBF**	cerebral blood flow
**CBV**	cerebral blood volume
**CMOS**	complementary metal-oxide semiconductor
**CPAP**	continuous positive airway pressure
**CW**	continuous-wave
**DAQ**	data acquisition
**DCS**	diffuse correlation spectroscopy
**DDS**	direct digital synthesizer
**DOS**	diffuse optical spectroscopy
**DOT**	diffuse optical tomography
**DSS**	digital synthesis system
**ECMO**	extracorporeal membrane oxygenation
**EEG**	electroencephalography
**EEL**	edge-emitting laser
**FD**	frequency-domain
**fNIRS**	functional NIRS
**FDPM**	frequency domain photon migration
**FPGA**	field-programmable gate array
**GSPS**	gigasample per second
**HbO**	oxy-hemoglobin
**HbR**	deoxy-haemoglobin
**HBT**	heterojunction bipolar transistor
**HbT**	total hemoglobin

**IC**	integrated circuit
**KNN**	k-nearest neighbor
**LUT**	look-up table
**MOSFET**	metal oxide silicon field effect transistor
**MSPS**	megasamples per second
**NEP**	noise equivalent power
**NIR**	near infrared
**NIRS**	near-infrared spectroscopy
**OEF**	oxygen extraction fraction
**$PbtO_2$**	cerebral tissue oxygen tension
**PCB**	printed circuit board
**PD**	photodiode
**PDC**	phase-to-digital converter
**PLL**	phase-locked-loop
**PMT**	photo-multiplier tube
**$rcSO_2$**	cerebral oxygen saturation
**RF**	radiofrequency
**SCM**	sternocleidomastoid
**SDS**	source-detector separation
**SiGe**	silicongermanium
**SiPM**	silicon photomultiplier
**SNR**	signal-to-noise ratio
**SOC**	system on a chip
**SPAD**	single-photon avalanche diode
**tDCS**	transcranial direct current stimulation
**TD-NIRS**	time-domain NIRS
**TIA**	transimpedance amplifiers
**TOBI**	tomographic optical breast imaging
**TTL**	transistor–transistor logic (missing)
**VCSEL**	vertical-cavity surface-emitting laser
**VGA**	variable gain

Table S1. Sources and Detectors Employed in Reviewed FD-NIRS Technologies.

Author, Year	Source Brand	Source Type	Part No. of Source	Light density of Source (mW)	Size of Source	Power consumption of Source	Detector Brand	Detector Type	Part No. of Detector	Photo-sensitive area of Detector	Size of Detector	Power consumption of Detector	Wavelength
Campbell et al., 2020 [21]	-	LD	-	-	-	-	Hamamatsu	APD	S12060-10	Φ1 mm	Φ5.4 × 3.7 mm^2	-	631, 660, 689, 782, 828, 849 nm
Choe et al., 2009 [23]	-	LD	-	-	-	-	-	APD	-	-	-	-	681, 783, 823,850 nm
Zhang,et al., 2005 [24,25]	-	LD	-	-	-	-	Hamamatsu	APD	C5331-04	Φ3 mm	80 × 45 × 19.6 mm	-	
El-Ghussein et al., 2013 [27]	-	LD	-	2-5	-	-	Hamamatsu	PMT	H9305-3	3.7 mm × 13 mm	53.2 × 50.8 × 19 mm^3	126 mW	660, 735, 785, 808, 826, 849 nm
Carp et al., 2017 [29]	-	LD	-	14, 10	-	-	Hamamatsu	PMT	R9880U-01	Φ8mm	Φ16 × 12.4 mm^2	-	670, 690, 700, 730, 760, 780, 810, 830 nm
Mackey et al., 2020 [30]	-	LD	-	-	-	-	-	PMT	-	-	-	-	-
Lee et al., 2022 [31]	-	-	-	-	-	-	-	-	-	-	-	-	785, 824 nm
Huang et al., 2017 [32]	Amplitude System	Yb-YAG laser	Mikan	-	-	-	Hamamatsu	APD Module	C5658	Φ0.5mm/ Φ3mm(in vivo)	28 × 50 × 60 mm^2	1.2W	700, 750, 800, 1030 nm
Zimmermann et al., 2016 [33]	Thorlabs	LD	HL6750MG/ HL8338MG	12.5	-	50 mW	Hamamatsu	APD Module	C5331-04	Φ3 mm	80 × 45 × 19.6 mm^3	-	685, 830 nm
Thompson et al., 2021 [34]	Thorlabs	LD	HL6738MG/ L852P50	12.5	Φ5.6 × 2.3mm	-	Thorlabs	APD Module	APD430 A	Φ0.5 mm	50.8 × 63.5 × 25.4 mm^3	3 mW	690 nm, 852 nm
Wathen et al., 2021 [36]	Thorlabs	LD	HL6738MG/ L852P50	10	Φ5.6 × 2.3mm	-	onsemi	SiPM	MicroRB-10020	Φ0.5 mm	50.8 × 63.5 × 25.4 mm^3	3 mW	690, 852 nm
Chen et al., 2004 [40]	-	LD	-	-	-	-	Hamamatsu	APD	S3884	Φ1.5 mm	Φ9.1 × 4.7mm^2	-	660, 780, 830nm
O'Sullivan et al., 2017 [42]	Vixar/Thorlabs	VCSEL / EEL	(VCSEL)/LD 808-SA60	11.6-17.8	-	155(Array)/143 (Single) mW	Hamamatsu	APD	S6045-05	Φ3 mm	Φ9.1 × 4.2 mm^2	-	680, 795, 850 nm
Torjesen et al., 2017 [46]	Thorlabs	LD Module	LDM9T	50	78.5 × 73.3 × 45.5 mm	12 W	Hamamatsu	APD Module	C5658	Φ0.5 mm/ Φ3 mm (in vivo)	28 × 50 × 60 mm^3	1.2 W	658, 690, 785, 808, 830, 850 nm

Istfan et al., 2019 [50]	Vixar	VCSEL	(Custom)	1.2-7.84	Φ5 mm	-	Hamamatsu	Compact APD	S13282-01CR	Φ0.2 mm	5.3 × 3.0 × 1.2 mm^3	-	660, 680, 775, 795 nm
Applegate et al., 2021 [51]	-	LD	-	-	-	-	Hamamatsu	APD	S11519-30	Φ3mm	Φ13.9 × 4.9 mm^2	-	690, 730, 785, 808, 830, 850 nm
Stillwell et al., 2021 [58]	Thorlabs/ Vixar	EEL/VCSEL	HL6738MG, L785P090, L808P200, L850P030, L980P030	35-100 (Factory)	Φ5.6 × 3.5 mm^2 (EEL)	240mW (EEL)	Hamamatsu	APD	S2384	Φ3mm	Φ9.1 × 4.2 mm^2	-	690, 785, 808,850, 940 980 nm
Miao et al., 2018 [63]	Thorlabs	LD	HL8338MG/ L785P090/H L6750MG	2.5	Φ5.6×2.3mm^2 for all	-	Hamamatsu	APD	S9251-15	Φ1.5mm	Φ9.1 × 4.2 mm^2	-	685, 785, 830 nm
Kılıç et al., 2022 [64]	Thorlabs	LD	HL6750MG/ HL8338MG	-	-	50 mW	Hamamatsu	APD	S9251-15	Φ1.5mm	Φ5.4 × 3.6 mm^2	-	690, 830 nm
Yazdi et al., 2017 [65]	BlueSky Research/Thorlabs/ Qphotonics	LD	VPSL0690-035X5A, VPSL0830-050X5A/L785P090/QLD-980-150S	35-50, (Factory)	Φ5.6×3.5mm^2 (Thorlabs)	240 mW	Hamamatsu	APD	S12023-02	Φ0.2mm	Φ5.4 × 3.7 mm^2	-	690, 785, 830, 980 nm
Chen et al., 2022 [66]	Thorlabs	LD	HL6750MG	-	-	50 mW	Hamamatsu	APD	C12703	Φ1.5mm	80 × 50 × 21.6 mm^3	-	685 nm
Koh et al., 2022 [67]	-	VCSEL	-	-	-	-	-	APD Module	-	-	-	-	-
Scammon, 2022 [68]	Thorlabs	LD	L785P25	25 (Factory)	Φ5.6 × 2.3mm	-	Thorlabs	APD Module	APD430 A	Φ0.5 mm	50.8 × 63.5 × 25.4 mm^3	3 mW	785 mm

Table S2. Phase Measurement Components Employed in Reviewed FD-NIRS Technologies.

Author, Year	Brand	Name	Part Number	Components Frequency Range	Number of Measurement channels	Size of Component
Campbell et al., 2020 [21]	Agilent	Network Analyzer	8753ES	30kHz-3GHz	1	222 mm × 425 mm × 457 mm
Choe et al., 2009 [23]	-	-	-	-	-	-
Zhang et al., 2005 [24,25]	-	I/Q Demodulator with PC	-	-	-	-
El-Ghussein et al., 2013 [27]	National Instruments	PC with signal down converted (With DAQ)	PCI 6031E	-	-	DAQ, 307 × 254 × 43 mm
Carp et al., 2017 [29]	-	PC with signal down converted	-	-	-	-
Mackey et al., 2020 [30]	-	PC with signal down converted	-	-	-	-
Lee et al., 2022 [31]	-	PC with signal down converted	-	-	-	-
Huang et al., 2017 [32]	Stanford Research	Lock-in Amplifier with signal down converted	SR830	1 MHz	1	-
Zimmermann et al., 2016 [33]	Xilinx	FPGA (With PC)	Spartan-6 LX9/(MATLAB)	-	1	FPGA, < 20mm × 20 mm
Thompson et al., 2021 [34]	-	PC with signal down converted	-	-	12	-
Wathen et al., 2021 [36]	-	PC with signal down converted	-	-	32	-
Chen et al., 2004 [40]	-	PC with signal down converted	-	-	-	-
O'Sullivan et al., 2017 [42]	-	Network analyzer	-	50 - 500 MHz	-	-
Torjesen et al., 2017 [46]	MircoZed	FPGA SoC Platform (With PC)	Xilinx Zynq-7010/(MATLAB)	-	1	FPGA, 10×6cm^2
Istfan et al., 2019 [50]	Agilent	Network Analyzer	E5061B	100 kHz - 3 GHz	1	-
Applegate et al., 2021 [51]	MircoZed	FPGA SoC Platform (With PC)	Xilinx Zynq /(MATLAB)	-	-	-
Stillwell et al., 2021 [58]	Krtkl Snickerdoodle	FPGA SoC Platform	Xilinx Zynq 7020	-	1	FPGA, 88.9×50.8 mm
Miao et al., 2018 [63]	-	130nm Custom Chip with PC	-	80MHz	1	2.25 mm^2
Kılıç et al., 2022 [64]	-	PC with signal pre-processing on custom chip	-	80MHz	1	-
Yazdi et al., 2017 [65]	-	180nm Custom chip	-	50-1000MHz	1	~5 × 5 mm
Chen et al., 2022 [66]	-	180nm Custom chip	-	10MHz	1	-
Koh et al., 2022 [67]	-	180nm Custom chip	-	150MHz	1	FD part, 2.5mm × 2.5 mm
Scammon, 2022 [68]	Xilinx	FPGA SoC Platform (With PC)	ZCU102	-	1	-
